# More than Marriages: How Nepal’s Marriage Equality Law Shape Public Health?

**Published:** 2024-12-14

**Authors:** Kamal Gautam, Kiran Paudel, Jeffrey A. Wickersham, Roman Shrestha

**Affiliations:** 1 Department of Allied Health Sciences, University of Connecticut, Storrs, CT 06269, USA; 2 Nepal Health Frontiers, Tokha 5, Kathmandu, Nepal; 3 Department of Internal Medicine, Section of Infectious Diseases, Yale School of Medicine, New Haven, CT, USA

**Keywords:** Marriage equality, LGBTIQA+, Public health, Health policy

## Abstract

Nepal became the second country in Asia to provide legal recognition of marriage to LGBTIQ+ couples after Taiwan. Moving beyond mere “same-sex marriage” to marriage equality is crucial, ensuring equal legal rights and opportunities for all when it comes to marriage. Studies indicate such legalization leads to positive health outcomes, including reduced STIs, lower rates of suicidal behavior, and decreased healthcare costs among LGBTIQA+. To fully benefit from the legal recognition of marriage to LGBTIQ+ couples, Nepal must align all laws, implement inclusive policies, and provide high-quality healthcare tailored to the needs of the LGBTIQA+.

## Tweetable abstract

Inclusive policy and high quality health service should be tailored to fulfill the needs of the LGBTIQA+.

## Background

Lesbian, Gay, Bisexual, Transgender, Intersex, Queer, and Asexual (LGBTIQA+) individuals have faced longstanding discrimination and marginalization globally. However, there’s been some progress in recent years, including in Nepal, with legislative protections directly in the Constitution. Despite this, the translation of the legislative protections on the ground faces challenges because of policy gaps, unclear definitions, and weak enforcement, especially concerning legal gender recognition for transgender people [1, 2]. Additionally, societal stigma and discrimination continue to hinder full acceptance and rights for LGBTIQA+ individuals in accessing essential services, such as health, education, and employment opportunities.

Against this backdrop, the Supreme Court of Nepal issued an interim order on June 27, 2023, which enabled the registration of marriage of LGBTIQ+ couples seeking legal recognition in the nation for the first time. This transformative step, following a writ placed on June 7, resulted in the registration and issuance of the first marriage certificate for transgender women (legally recognized as male) and cisgender men on November 29, 2023 [3]. Nepal became only the second country in Asia to give legal recognition of marriage after Taiwan- a huge step toward marriage equality in the entire Asia-Pacific region and a model for other countries to follow. As we celebrate this landmark decision, it is imperative to understand that its implications extend beyond the legal sphere, encompassing substantive consequences for the public health of LGBTIQA+ in Nepal.

### Framing the discussion

The deliberate use of the term marriage equality instead of “same-sex marriage” aims to shape the conversation in a way that emphasizes equal rights and inclusivity. The term marriage equality conveys the idea that all individuals, regardless of their diverse sexual orientation, gender identity, gender expression and sex characteristics, should have the same legal rights and opportunities when it comes to marriage.

### Positive health outcomes from marriage equality

An emerging body of literature has consistently indicated positive health benefits of marriage equality. In countries where marriage equality has been codified, there has been a reduction in sexually transmitted infections [4, 5], lower youth suicide attempts [6], and decreased psychiatric and alcohol use disorders [7] among sexual minority individuals following the legalization of marriage equality.

Similarly, laws that support marriage equality in the United States have shown notable decreases in medical care visits, mental health care visits, and associated costs among gay and bisexual men, and men who have sex with men [8]. Furthermore, in Taiwan, the legalization of same-sex marriage was linked with a decrease in depressive symptoms among sexual minority men [9]. Similarly, a study in California found that gay, lesbian, and bisexual adults in same-sex marriages experienced lower levels of psychological distress compared to their counterparts who were not married [10]. Additionally, in the United States, where marriage equality has been legalized, spousal benefits have significantly enhanced healthcare access for same-sex couples by providing them with greater access to employer-sponsored health insurance and healthcare services [11].

The positive health outcomes of marriage equality could largely be attributed to its role as an anti-discrimination policy that promotes societal acceptance and inclusivity for LGBTIQA+ individuals and couples [12, 13]. By fostering an environment of actual or perceived social validation, marriage equality could enhance a sense of belonging and security among LGBTQ+ couples. This shift not only encourages inclusivity but also significantly improves access to healthcare through spousal benefits, thereby addressing both physical and mental health needs more effectively.

The recent recognition of marriage equality in Nepal holds great promise for improving the alarming mental health crisis among LGBTIQA+, while also enhancing general healthcare access [14–17]. This legislation not only promotes equality but also fosters social acceptance, increasing a sense of belonging and security which facilitates improved healthcare through spousal benefits. Additionally, family-based health insurance plans offered at a low cost by the government could result in expanded access to healthcare for LGBTIQA+ individuals when they enter marriage.

### Way forward

Nepal must take proactive steps towards creating an inclusive legal and social framework to fully harness potential health benefits and uphold the principles of equality. First, a crucial initial step involves amending existing discriminatory laws, such as Civil Code Chapter 3 Section 67, which defines marriage as a union between a “Male and Female” under family law [18]. Aligning these statutes with the Supreme Court’s recent decision is imperative not only to safeguard the legal rights of LGBTIQA+ but also to positively impact their overall health and well-being.

Second, Nepal must swiftly implement and enforce policies and programs that guarantee access to gender-affirming care, such as hormone therapy, testing, and necessary support for gender-related surgery. By doing so, Nepal can ensure that LGBTIQA+ receives the healthcare required, fostering a healthier and more supportive environment.

Third, Nepal must develop clear laws and policies that grant surrogacy, adoption, and parental rights to all couples, irrespective of their identity, to ensure equitable family building opportunities.

While a change in law is paramount, it is essential to note that challenges and disparities may persist, and the broader societal context plays a crucial role in determining the full impact of such legal developments. Fourth, the government should strengthen and enforce anti-discrimination laws within the health care system to ensure that everyone receives equal, respectful, and compassionate care. Simultaneously, civil society organizations should continue their advocacy efforts for the enactment and enforcement of policies at all levels (local, provincial, and federal) to maximize the health and social benefits associated with marriage equality. Nepal’s journey to become a global LGBTIQA+ health and rights beacon starts by putting these inclusive laws and policies into action.
